# Supplementary material for: Transcriptomic profile of the hippocampus of rat strains with contrasting nervous system excitability
Source: PLoS One. 2026 Jun 3;21(6):e0350674. doi: 10.1371/journal.pone.0350674 (PMC13232848; doi:10.1371/journal.pone.0350674)
Supplement: S1 Fig — (DOCX) [file pone.0350674.s001.docx]

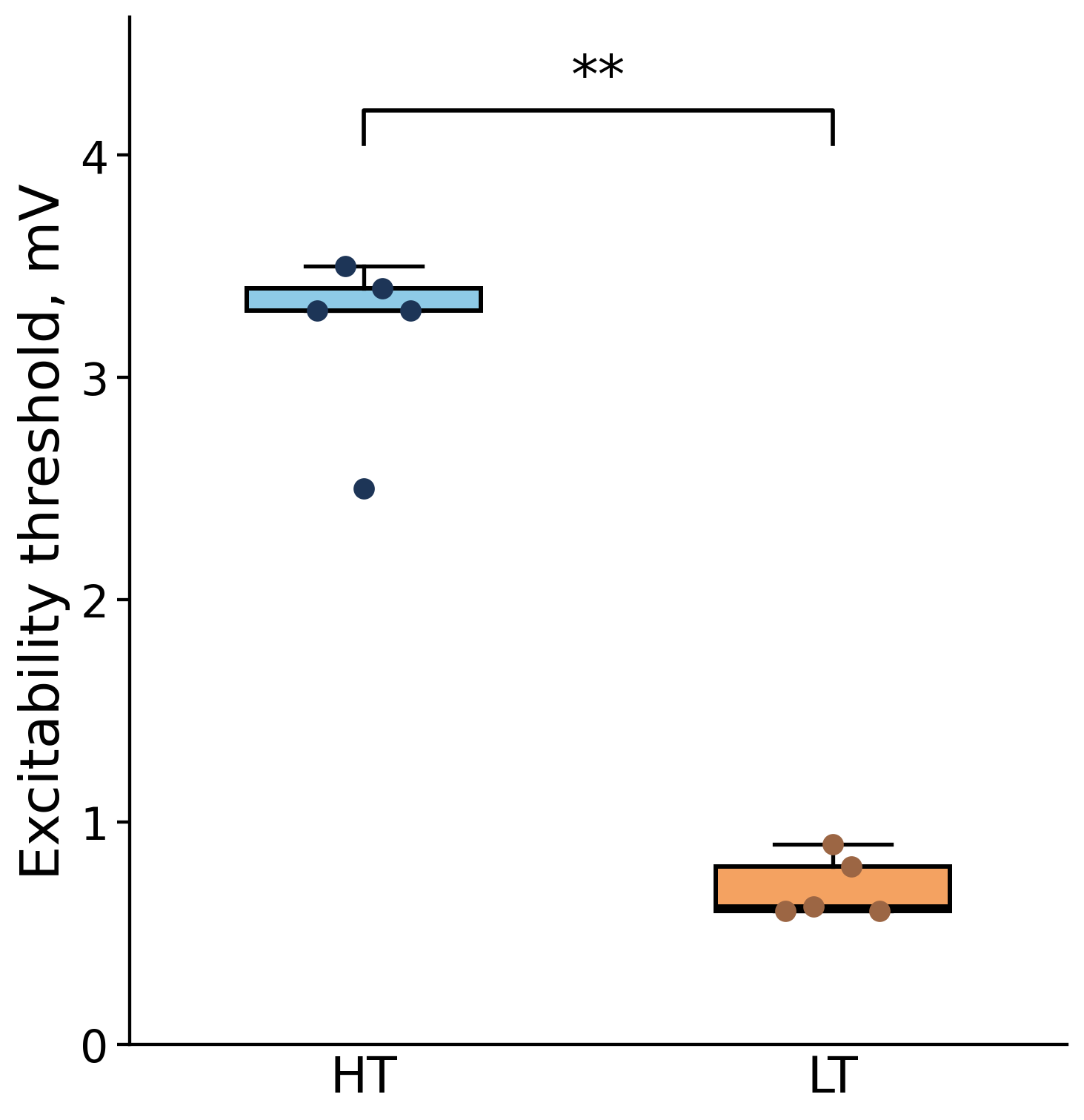


**Supplementary Figure 1. Individual excitability thresholds in selectively bred HT and LT rats.**
Neuromuscular excitability thresholds were measured in high-threshold (HT) and low-threshold (LT) rat ыекфшты. Each point represents an individual animal. Box plots show the median and interquartile range. Mann–Whitney U test. **p < 0.01.
