# Supplementary material for: Transcriptomic profile of the hippocampus of rat strains with contrasting nervous system excitability
Source: PLoS One. 2026 Jun 3;21(6):e0350674. doi: 10.1371/journal.pone.0350674 (PMC13232848; doi:10.1371/journal.pone.0350674)
Supplement: Attachment — Submitted filename: Response.docx [file pone.0350674.s012.docx]

Editor

We noticed you have some minor occurrence of overlapping text with the following previous publication(s), which needs to be addressed:

[https://doi.org/10.1371/journal.pone.0323325](https://track.editorialmanager.com/CL0/https:%2F%2Fdoi.org%2F10.1371%2Fjournal.pone.0323325/1/010f019cff195c75-ff02f6ae-53aa-469e-9bd8-d6a69bd08434-000000/4frULco43gHSyjtgbuZ9HMf18EM9pI1I29Ns2FeqMx8=254)

In your revision ensure you cite all your sources (including your own works), and quote or rephrase any duplicated text outside the methods section. Further consideration is dependent on these concerns being addressed.

We re-examined the revised manuscript for any duplicated wording outside the Methods section, including overlap with our own previously published work, and corrected the passages that could be considered too close in phrasing. Most of the overlap was limited to short, highly conventional technical or descriptive phrases, mainly in figure legends and brief transition sentences in the Results section. Wherever possible, we rephrased these passages to avoid unnecessary textual similarity.

4. Please note that funding information should not appear in any section or other areas of your manuscript. We will only publish funding information present in the Funding Statement section of the online submission form. Please remove any funding-related text from the manuscript.

Done

5. Please ensure that you refer to Figure 6 in your text as, if accepted, production will need this reference to link the reader to the figure.

Done

6. Please upload a new copy of Figures 5 and 6 as the detail is not clear. Please follow the link for more information:  [https://journals.plos.org/plosone/s/figures](https://track.editorialmanager.com/CL0/https:%2F%2Fjournals.plos.org%2Fplosone%2Fs%2Ffigures/1/010f019cff195c75-ff02f6ae-53aa-469e-9bd8-d6a69bd08434-000000/ESZI4KhI9vVIxhFq3mQzBHPwVLNO5PBUDEPUjYflHjE=254)

Done

7. We are unable to open your Supporting Information file ( Supplementary_1 string_MCL_2_GO_1.tsv and Supplementary_2 string_MCL_2_GO_6.tsv) Please kindly revise as necessary and re-upload.

We have revised the Supporting Information files and converted the supplementary tables, including Supplementary_1 string_MCL_2_GO_1 and Supplementary_2 string_MCL_2_GO_6, into Excel to ensure they can be opened without technical difficulty.

The two reviewers raised several concerns about methodologies that are presented without enough details notably regarding animal welfare, description and justification of the selected model, and for the GO analysis. Furthermore, they found that PPI analysis does not reflect an independent layer of experimental evidence from the present study. Finally, it is not clear why the differential gene expression analysis has been performed with a log2FoldChange filter >0.379 and not >1 as classically used and how it may impact the conclusion. Although, I personally think that your manuscript has some merits, I invite you to address those important issues during revision.

We believe that these points have been addressed in the revised manuscript. In response to the reviewers’ comments, we clarified the animal welfare reporting, improved the description and justification of the selective breeding model, revised the GO analysis section for greater methodological transparency and interpretive caution, strengthened the PPI analysis by connecting network properties with differential expression metrics, and clarified the rationale for the selected log2FoldChange threshold.

**Reviewer #1:  Main comments**
- The transcriptomic study fails to count with readouts of another part of the brain that could be considered as non-relevant for the excitability response, thus working as a negative control for potential differences among animals.

We agree that analysis of an additional brain region could help to assess regional specificity more fully. However, in this model, another brain area cannot be considered a true negative control, because inherited differences in nervous system excitability may affect multiple brain structures. At the same time, our previous transcriptomic study of the amygdala (Shalaginova, 2025 in References) showed a different pattern, with a much stronger immune-related signature, although the amygdala is also involved in stress-related regulation. This supports the view that the observed changes are not simply uniform brain-wide differences, but may vary across regions.

- Surprisingly, the differential gene expression analysis has been performed with a log2FoldChange filter >0.379…i.e. that the authors conserve/consider as differentially expressed genes those that have a fold-change of at least 1.3. This threshold appears quite mild. It would be interesting to know how many genes do pass a log2FoldChange filter >1; which is more classically used. Could such more stringent threshold still conserve the differences described by the authors?
-
We thank the reviewer for this important comment regarding the stringency of the criteria used to select differentially expressed genes when assessing inter-strain differences. We have now clarified that |log2FC| > 0.379 was not used as the primary criterion for DEG detection. Differential expression was defined primarily by FDR-adjusted significance (padj < 0.05), whereas the |log2FC| > 0.379 filter was applied only for visualization in the circular heatmap to reduce the number of displayed genes and highlight those with more pronounced between-strain differences.

The choice of a moderate fold-change threshold was based on the biological context of the study. The aim of our study was to compare the transcriptomic profiles of intact rats from selectively bred strains contrasting in nervous system excitability under normal physiological conditions. In this context, central regulatory mechanisms are expected to operate within the limits of homeostatic stability, supported by the evolutionary conservation of molecular and transcriptional processes in the brain. Therefore, large two-fold changes in gene expression are not necessarily expected, whereas moderate but statistically robust expression shifts may still be biologically meaningful. More stringent thresholds such as |log2FC| > 1 are useful for identifying large-amplitude transcriptional responses and may be particularly appropriate in models of severe brain injury, stress exposure or pathology.

We believe that using that using of this approach to the criterion for selecting DEGs allows us to retain genes with less pronounced but potentially biologically meaningful expression differences, as well as the pathways with which they are associated. This approach may provide insight into more subtle mechanisms regulating brain function under selective breeding for nervous system excitability and thereby enrich the interpretation of the results. Methodological studies in RNA-seq analysis have cautioned that stringent fold-change filtering may remove genes with genuine differential expression, particularly when expression changes are moderate but consistent across samples (Anqi Zhu, et al, Heavy-tailed prior distributions for sequence count data: removing the noise and preserving large differences, Bioinformatics, Volume 35, Issue 12, June 2019, Pages 2084–2092, https://doi.org/10.1093/bioinformatics/bty895).

To further support this rationale, we cite several neurotranscriptomic studies in which differential expression analyses were performed using low fold-change thresholds, including thresholds around 0.3 or without an explicit fold-change cutoff. This suggests that mild log2FC filtering is an accepted analytical option in neuroscience when the aim is to detect coordinated, moderate transcriptional differences rather than only large-magnitude expression changes.

- Goh JY, et al. Transcriptomic analysis of rat prefrontal cortex following chronic stress induced by social isolation - Relevance to psychiatric and neurodevelopmental illness, and implications for treatment. Neurobiol Stress. 2024 Oct 17;33:100679. doi: 10.1016/j.ynstr.2024.100679
- Chen MB, et al. Persistent transcriptional programmes are associated with remote memory. Nature. 2020 Nov;587(7834):437-442. doi: 10.1038/s41586-020-2905-5. Epub 2020 Nov 11. Erratum in: Nature. 2025 Aug;644(8077):E37. doi: 10.1038/s41586-025-09463-4
- Ayhan F, et al. Resolving cellular and molecular diversity along the hippocampal anterior-to-posterior axis in humans. Neuron. 2021 Jul 7;109(13):2091-2105.e6. doi: 10.1016/j.neuron.2021.05.003
- Mathys H, et al. Single-cell transcriptomic analysis of Alzheimer's disease. Nature. 2019 Jun;570(7761):332-337. doi: 10.1038/s41586-019-1195-2
- Makovka YV, et al. Effect of Short-Term Restraint Stress on the Expression of Genes Associated with the Response to Oxidative Stress in the Hypothalamus of Hypertensive ISIAH and Normotensive WAG Rats. Antioxidants (Basel). 2024 Oct 26;13(11):1302. doi: 10.3390/antiox13111302

As suggested by the reviewer, we additionally applied a more stringent |log2FC| > 1 threshold. This resulted in 38 genes, now listed in Supplementary Table 1. GO enrichment analysis of this restricted gene set produced only a small number of enriched terms, many of which were weakly related to nervous system function. Thus, applying a stringent |log2FC| > 1 threshold would address a different question: which genes show large-amplitude expression changes. Whereas our study aimed to characterize the functional organization of moderate but coordinated inter-strain differences in gene expression.

The discussion part contains several elements that should be retrieved in the results section.

We revised the manuscript to improve the separation between the Results and Discussion sections. Specifically, we moved or condensed several result-like summary elements that had previously appeared in the Discussion, especially where the text described functional groupings of DEGs, enriched GO categories, or the structure of PPI clusters.

Minor comments

- The presented figures are not ordered, and their quality are quite low. Fixed
- Figure 5&6 are missing color legends for edges and nodes. Fixed

**Reviewer #2:  Main comments**
1. PLOS ONE requires that manuscripts reporting animal research explicitly state, within the Methods section, the steps taken to ameliorate animal suffering. As currently written, the Methods do not adequately address this requirement. Specifically, the described euthanasia procedure — decapitation by guillotine without prior anaesthesia and in the absence of explicitly defined humane endpoints — appears difficult to reconcile with the requirements of the current European Directive 2010/63/EU, which mandates explicit humane endpoints and imposes stricter conditions on the justification of painful or distressing procedures than its predecessor, Directive 86/609/EEC.

We re-examined this point in light of the current European Directive 2010/63/EU. We note that the Directive requires that methods of euthanasia should minimize pain, suffering, and distress, and that the appropriate species-specific method should be used. Also, decapitation in rodents is treated as a method to be used when other approaches are not suitable in the specific experimental context. In our study, the terminal procedure was selected on the basis of harm-benefit balance and endpoint validity. The primary outcome was transcriptomic analysis of immune- and cytokine-related signals in intact hippocampal tissue. Inhalational anesthesia such as isoflurane can rapidly alter pro-inflammatory gene expression and therefore would directly compromise the main molecular readout of the study. Under these conditions, the use of prior anesthesia would reduce the scientific validity of the terminal sampling procedure and could lead to animal use without obtaining interpretable data. We have clarified this rationale in the revised Methods and have expanded the description of measures taken to minimize suffering, including routine welfare monitoring, minimization of handling time, and rapid performance of the terminal procedure by experienced personnel.

2. The authors should expand their description and justification of the selective breeding model. A previous publication by Vylegzhanina and colleagues analysed rat strains with contrasting excitability levels across approximately 70 generations, whereas the present manuscript states that interstrain differences stabilised at the 10th generation, at which point between-strain variability exceeded within-strain variability by more than fourfold. This apparent discrepancy is not discussed, and the relationship between the earlier long-term breeding programme and the present cohort is unclear. The authors should explicitly clarify the generational history of the strains used here, discuss how phenotypic stability was monitored across generations, and situate the present cohort within the broader longitudinal context of the model. To this end, it would be highly valuable to include a figure or supplementary table illustrating the trajectory of excitability threshold divergence and within-strain variance across at least the first ten generations of selection, as this would provide readers with the empirical basis for the claim of phenotypic stabilisation and strengthen confidence in the genetic model underlying the transcriptomic comparisons.

Thank you for this important comment. We agree that the wording in the original version was not sufficiently clear and could create confusion regarding the generational history of the model. The statement that interstrain differences had stabilized by the 10th generation refers to the early phase of the selective breeding program, that is, the point at which divergence between the strains became clearly established and exceeded within-strain variability by more than fourfold. It does not refer to the generation used in the present experiment. The animals studied in the current study belonged to the 76th generation of the long-term breeding program.

We have clarified this explicitly in the revised manuscript. We have also added text explaining that nervous system excitability was monitored both during the selective breeding process, when animals were chosen for further breeding, and during the formation of experimental groups. In addition, we have included supplementary material showing individual excitability thresholds, to better place the present cohort within the broader context of the model (Supplementary Figure 1).

3. The Results section would benefit from expansion and improved internal signposting. At present, the transition between analytical stages — from differential expression to GO enrichment to PPI network analysis — is abrupt, and summary statements foregrounding the key findings of each subsection are largely absent. Adding brief transitional paragraphs that synthesise the reasoning and highlight the principal outcomes of each analytical layer would substantially improve readability and logical flow.

In the revised manuscript, we added brief transitional and summary sentences to improve the logical flow between differential expression analysis, functional enrichment, and PPI analysis.

Furthermore, the RNA sequencing data generated in this study do not appear to have been deposited in a public repository. Deposition in an appropriate archive (e.g., NCBI GEO or ArrayExpress) prior to publication is strongly encouraged and is consistent with open-science standards increasingly required by journals in this field. The accession number should be reported in the Methods section.

We deposited the raw and processed data in NCBI and added the corresponding link in the Methods section. (GSE327807).

4. The Discussion makes repeated cell-type-specific interpretive claims that are not supported by the bulk sequencing methodology employed. Attributing differences in Gfap expression to astrocytic reactivity, Slc2a5 to microglial metabolic state, P2rx7 to glial purinergic signalling, and Stab2 to an endothelial or myeloid neuroimmune profile implies a cellular resolution that bulk hippocampal RNA-seq fundamentally cannot provide. While the authors appropriately acknowledge in the Conclusion that their findings "motivate testable hypotheses to be evaluated in follow-up studies," this caveat is insufficiently foregrounded in the body of the Discussion, where speculative inferences frequently appear without explicit epistemic qualification. The authors are encouraged to systematically reframe cell-type-specific and mechanistic claims as hypotheses, accompanied in each case by a brief statement of what additional evidence — such as single-nucleus RNA sequencing, cell-type-resolved proteomics, or targeted immunohistochemistry — would be required to substantiate them.

In the revised manuscript, we systematically softened such statements, reframed them as hypothesis-generating where appropriate, and added explicit clarification that bulk hippocampal RNA-seq does not provide direct cell-type resolution. We also indicated that these interpretations would require validation by additional approaches such as single-cell RNA sequencing, deconvolution or immunohistochemistry.

5. The GO enrichment analysis is presented without adequate methodological detail or critical evaluation of its inherent limitations. The background gene set used is not reported, precluding reproducibility assessment. More substantively, terms such as "cognition," "learning and memory," and "regulation of synaptic plasticity" are among the most annotation-dense in GO databases and are prone to enrichment in virtually any transcriptomic dataset derived from neural tissue; their appearance cannot be interpreted as strong evidence of pathway-specific divergence without further qualification. The authors should report all enrichment parameters, apply a redundancy-reduction approach (e.g., semantic clustering via REVIGO or equivalent) to present a non-redundant summary of enriched terms, and explicitly distinguish high-specificity terms — which carry stronger inferential weight — from broad, annotation-rich terms that may reflect database structure as much as biology.

Substantial additions have been made to the Methods and Results sections. We have added the background gene set (Supplementary Table 1) used for GO enrichment analysis as a supplementary file to ensure transparency and reproducibility of the enrichment procedure.

We agree that broad GO terms such as “cognition,” “learning and memory,” and “regulation of synaptic plasticity” are highly annotation-dense and should not be interpreted as strong evidence of pathway-specific divergence on their own. We have revised the text to avoid overinterpretation of these broad categories.

At the same time, we would like to note that the enrichment of these terms in the hippocampal dataset was not treated as the sole basis for our conclusions. The interpretation was based on their co-occurrence with more specific GO terms and gene-level changes related to synaptic organization, vesicle trafficking, neuronal signaling, and glial/vascular regulation. Moreover, in our parallel analysis of the amygdala from the same animals under the same experimental conditions, GO enrichment was dominated mainly by immune- and inflammation-related processes rather than by cognition- or plasticity-related terms (Shalaginova et al., 2025 in References). This comparison suggests that the hippocampal enrichment profile is not merely a generic consequence of analyzing neural tissue, although we agree that broad GO terms require cautious interpretation.

6. The protein-protein interaction network analysis using the STRING database with the discussion of individual cluster nodes — including SNAP25, NTRK2, MAPT, GFAP, AGT, FLT1, and PDGFRB — focuses primarily on their established biological roles as reported in the broader literature, rather than on their network-level properties (e.g., degree, betweenness centrality) or their specific quantitative contribution to interstrain transcriptional divergence. As a result, the PPI analysis functions largely as a thematically organised literature review rather than as an analytically independent layer of evidence. Connecting network topology explicitly to the magnitude of differential expression for each highlighted node would substantially strengthen the analytical value of this section.

In the revised manuscript, we strengthened this part in three ways. First, we added short result-level paragraphs describing the network structure more explicitly. Second, for the genes discussed in the Discussion section, we now report their node degree values and refer to their position within the corresponding network/module rather than discussing them only from the broader literature perspective. Third, we added a supplementary table integrating the STRING node-degree information with the DESeq2 results (Supplementary Tables 3 and 6), so that the network position of the highlighted nodes can be considered together with the magnitude and direction of inter-strain transcriptional differences.
