# Supplementary material for: Transcriptomic profile of the hippocampus of rat strains with contrasting nervous system excitability
Source: PLoS One. 2026 Jun 3;21(6):e0350674. doi: 10.1371/journal.pone.0350674 (PMC13232848; doi:10.1371/journal.pone.0350674)
Supplement: Attachment — Submitted filename: Response_auresp_2.docx [file pone.0350674.s013.docx]

Reviewer #3: 

1. PCA analysis
The current presentation of the PCA based solely on the 654 differentially expressed genes (DEGs) is methodologically problematic, as it introduces a circular analysis issue — also known as "double dipping": since genes were selected precisely because they differ between groups, the resulting separation and the variance explained by the first principal components are artificially inflated. Importantly, a PCA performed on all expressed and variable genes used as background for the DESeq2 analysis already achieves a clear and complete separation of the HT and LT groups into non-overlapping clusters, which constitutes a far more rigorous and compelling demonstration of transcriptomic divergence between the two conditions. The authors should therefore present the PCA based on all background genes as the primary analysis, and may retain the DEG-based PCA as a supplementary figure for comparison, clearly contrasting the variance explained in both cases and explicitly discussing the methodological distinction.

We thank the reviewer for this important methodological comment. We performed an additional PCA using the transcriptome-wide background expression matrix without DEG-based gene selection. All expressed genes were included after removal of genes with zero expression or zero variance across samples. The revised manuscript now presents both the DEG-based PCA (Fig. 2A) and the background-gene PCA (Fig. 2B). Importantly, clear separation between LT and HT samples was also preserved in the unbiased background PCA, although the variance explained by the first principal components was lower, as expected for a transcriptome-wide analysis not restricted to DEGs. The Methods and Results sections were revised accordingly.

2. Protein-protein interaction (PPI) enrichment analysis
The significant PPI enrichments reported for networks derived from GO biological process gene sets are fully expected by construction, as genes sharing a biological process annotation are inherently more likely to interact, and therefore these results add limited interpretive value to the manuscript. A more informative and meaningful observation would be to highlight that the full set of 654 DEGs (FDR < 0.05) itself shows significant PPI enrichment, which provides evidence that the transcriptional response to the experimental contrast is not a collection of independent gene-level changes, but rather reflects the perturbation of coordinated biological networks.
STRING network:
number of nodes: 639
number of edges: 547
expected number of edges: 381
PPI enrichment p-value: 7.77e-16
<https://version-12-0.string-db.org/cgi/network?networkId=bsNRxR3QJrRd>
This PPI enrichment should be presented as a key result, as it both strengthens the biological relevance of the findings and directly justifies the more focused PPI analyses conducted on the DEGs associated with the GO biological processes "regulation of the MAPK cascade" and "regulation of synaptic plasticity."

We thank the reviewer for this helpful suggestion and analysis. The Results section was revised to include this global PPI enrichment before the focused PPI analyses. The Discussion was also modified to interpret this result in the context of inherited nervous system excitability as a polygenic trait. The global STRING network was added as Supplementary Fig. 2 and node degrees as Supplementary Table 8.
